# Novel Europium-Grafted 3D Covalent Organic Framework for Selective and Sensitive Fluorescence-Enhanced Detection of Levofloxacin

**DOI:** 10.3390/s25072304

**Published:** 2025-04-04

**Authors:** Junyi Zhao, Chao Zhang, Zhijie Qiu, Zerong Zhang, Xiaorou Lin, Shibin Huang, Jianfeng Zhang, Jingpeng Wu, Li Liao, Rui Wang

**Affiliations:** 1 Northeast Guangdong Key Laboratory of New Functional Materials, School of Chemistry and Environment, Jiaying University, Meizhou 514015, China; junyi98@mail.ustc.edu.cn; 2 Hefei National Research Center for Physical Sciences at the Microscale, School of Physical Sciences, University of Science and Technology of China, Hefei 230026, China; 3 The Third Affiliated Hospital of Anhui Medical University (The First People’s Hospital of Hefei), Hefei 230001, China; chao.zhang@ahu.edu.cn; 4 Guangdong Weipu Testing Technology Co., Ltd., Guangzhou 510275, China; qzjieaa@gmail.com; 5 State Key Laboratory of Inorganic Synthesis and Preparative Chemistry, Jilin University, Changchun 130012, China; zerongzhang0314@gmail.com (Z.Z.); 202430700765@mail.scut.edu.cn (X.L.); shibinhuang666779@outlook.com (S.H.); zjf1930468484@outlook.com (J.Z.); wujingpeng17613@outlook.com (J.W.); 6 School of Chemical Engineering and Technology, College of Chemistry, GBRCE for Functional Molecular Engineering, IGCME, Sun Yat-sen University, Guangzhou 510275, China

**Keywords:** three-dimensional covalent organic framework, ***bcu*** net, lanthanide luminescence, levofloxacin sensing

## Abstract

Levofloxacin (LVFX), a fluoroquinolone antibacterial agent widely used in treating bacterial infections, poses significant risks when overused, necessitating the development of reliable and efficient detection methods. Herein, we introduce Eu@SUZ−103, a novel europium-grafted three-dimensional covalent organic framework (COF) featuring an eight-connected ***bcu*** net, for the selective detection of LVFX in serum and urine. Its 3D architecture facilitates rapid LVFX diffusion to luminescent sites, producing notably enhanced fluorescence and high sensitivity. Evaluations in complex biological matrices revealed excellent performance encompassing a broad linear range (5–2000 μM) and a low detection limit. Altogether, Eu@SUZ−103 extends the practical scope of 3D COFs in fluorescence-based sensing, offering a robust platform for accurate, efficient, and selective LVFX monitoring in clinical and environmental applications.

## 1. Introduction

Antibiotics have been indispensable in combating infectious diseases since the discovery of penicillin, and fluoroquinolones have emerged as particularly potent agents in this regard [1,2,3]; among these, levofloxacin (LVFX) is renowned for its efficacy against respiratory tract and soft tissue infections [4,5,6,7,8], yet the misuse or overuse of LVFX can trigger severe adverse effects such as gastrointestinal distress, neurological complications, and psychiatric symptoms [9,10,11], thereby highlighting the necessity of precise and efficient detection methods for both clinical and environmental matrices. Although liquid chromatography–tandem mass spectrometry (LC–MS), high-performance liquid chromatography (HPLC), spectrophotometry, and capillary electrophoresis (CE) remain the mainstream approaches for quantification of LVFX [12,13] and provide remarkable sensitivity along with low detection limits, these techniques frequently demand specialized equipment, experienced technicians, and occasionally toxic chemicals, thereby limiting their accessibility in settings where rapid and straightforward monitoring is essential. Against this backdrop, fluorescence-based sensing has garnered considerable attention for antibiotic detection by leveraging the variations in fluorescence emission when LVFX interacts with a suitable probe or sensor [14,15,16,17,18]; these changes, which can manifest in terms of intensity or emission wavelength, facilitate the measurement of LVFX concentrations without the extensive infrastructure or operational complexity typical of conventional methods. Therefore, establishing a practical and efficient fluorescence-based method for the detection of LVFX is of critical importance.

Europium ion (Eu^3+^) is a promising luminescent material for fluorescence-based detection due to the high purity of its emission color, narrow emission bands, and strong quantum yields, which make it particularly appealing for sensing various analytes [19,20,21,22,23,24], including levofloxacin (LVFX) [25,26]. However, its broad application is constrained by low absorption coefficients and weak luminescence associated with the parity-forbidden nature of f-f transitions, prompting researchers to employ organic ligands as “antennas” to absorb incident light and transfer energy efficiently to the central metal ion. Notably, with its β-diketonate structure, LVFX can significantly enhance Eu^3+^ emission as an effective antenna. Despite these advantages, Eu^3+^ complexes often suffer from poor light and thermal stability, limited mechanical robustness, and a tendency to aggregate, which undermine their reliability in complex detection scenarios for high-performance LVFX sensing.

Covalent organic frameworks (COFs) [27,28,29,30,31,32] are crystalline, porous materials formed by covalent bonds between organic building units, conferring high chemical stability and tunable porosity; these attributes make COFs highly versatile in areas such as energy storage, catalysis, metal ion detection, gas separation, and adsorption [33,34,35,36,37]. Recently, Yang et al. [38] synthesized Eu@COF, which was successfully employed for the “turn-off” detection of levofloxacin (LVFX). By anchoring lanthanide complexes to COFs, their stability is enhanced and aggregation is prevented. The intrinsic porosity of COFs also promotes efficient analyte diffusion, improving detection sensitivity [39,40,41,42]. In comparison to small-molecule fluorescent probes, COFs offer unique advantages for biological sensing applications. Their high chemical stability, customizable functionality and tunable porosity provide greater binding capacity and better analyte diffusion, translating into improved sensitivity. Although small-molecule probes may be soluble and simple to handle, they often suffer from limited structural adaptability, which restricts their effectiveness in complex environments such as serum or urine, where analyte concentrations may vary or be impacted by interference. Nevertheless, most reported COFs are two-dimensional [43], and creating three-dimensional (3D) COFs with ordered, expandable frameworks remains challenging. Compared with 2D COFs, 3D COFs possess larger surface areas, interconnected channels, and more accessible functional sites [44,45,46,47,48,49]. These features enhance their performance in fluorescence-based sensing, particularly by improving the efficiency of analyte diffusion and increasing the number of active sites available for interaction. In contrast, 2D COFs with planar structures tend to have limited accessibility to functional sites and are often constrained by lower surface areas, which can reduce their sensing efficiency. Additionally, the interconnected channels in 3D COFs facilitate the transport of analytes to the sensing sites, enhancing detection sensitivity and selectivity. However, only a limited number of 3D COFs, particularly those with novel topologies such as the eight-node connected bcu structure [50,51,52], have been synthesized. From a theoretical standpoint, grafting lanthanide metals onto these rare 3D COFs can stabilize lanthanide complexes, boost sensitivity, and broaden the scope of fluorescence-based antibiotic detection. However, substantial obstacles persist in implementing this strategy and to the best of our knowledge, Eu^3+^-grafted COFs featuring an eight-connected bcu net have not been reported so far.

Taking these considerations into account, we report for the first time the synthesis of Eu^3+^-grafted COFs featuring an eight-connected bcu net, designed for the selective detection of levofloxacin (LVFX) in serum and urine samples. These COFs, designated as SUZ−103 and Eu@SUZ−103 (SUZ stands for Sun Yat-sen University, Zhuhai), possess large channels, permanent porosity, high chemical stability, and excellent crystallinity. Eu@SUZ−103 exhibits significant LVFX-specific luminescence enhancement due to the efficient energy transfer between LVFX and the Eu@SUZ−103 framework. Additionally, the three-dimensional (3D) architecture of Eu@SUZ−103 facilitates the effective diffusion of LVFX analytes to the luminescent sites, ensuring extensive interaction with the active sensing regions. This efficient analyte transport, coupled with robust energy transfer mechanisms, enhances both the sensitivity and selectivity of LVFX detection. These structural and functional advantages enable Eu@SUZ−103 to accurately monitor LVFX in complex biological matrices, including serum and urine, while offering a wide linear range and a low limit of detection.

## 2. Materials and Methods

### 2.1. Materials

The materials and reagents involved in this work are described in the Supplementary Materials.

### 2.2. Synthesis of SUZ−103 and Eu@SUZ−103

**Scheme 1:**
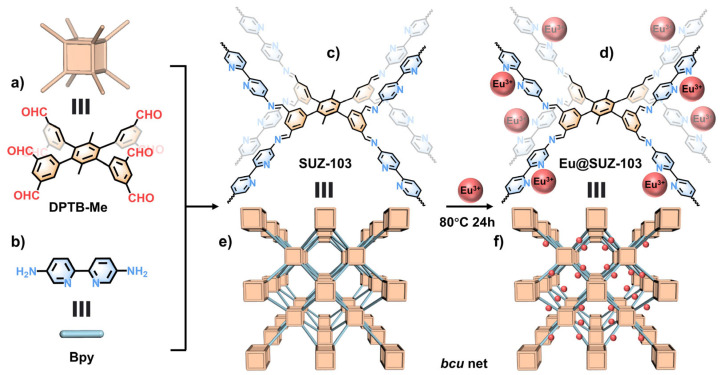
Molecular structures of (**a**) DPTB-Me with cubic (8-connected) knots. (**b**) Structure of a linear building block Bpy. Extended frameworks of (**c**) SUZ−103 and (**d**) Eu@SUZ−103. A ***bcu*** net for (**e**) SUZ−103 and (**f**) Eu@SUZ−103.

Our strategy for developing Eu^3+^-grafted COFs featuring an 8-connected bcu net involved the use of two monomers: 4′,5′-bis(3,5-diformylphenyl)-3′,6′-dimethyl-[1,1′:2′,1″]-terphenyl-3,3″,5,5″-tetracarbaldehyde (DPTB-Me) serving as a D_2_h-symmetric octatopic monomer, and 2,2′-bipyridine-5,5′-diamine (Bpy) chosen as a linear linker. As shown in Scheme 1, SUZ−103 (Scheme 1c) was synthesized via a Schiff base condensation reaction between DPTB-Me (Scheme 1a) and Bpy (Scheme 1b), producing extended 3D bcu nets (Scheme 1e). The synthesis of SUZ−103 was carried out using a traditional solvothermal approach by suspending DPTB-Me and Bpy in a mixed solvent of dioxane and mesitylene in the presence of acetic acid, followed by heating at 120 °C for 3 days. To construct the Eu^3+^-grafted COF, SUZ−103 was dispersed in an acetonitrile solution of Eu (acac)_3_ at 80 °C for 24 h. The product was separated by centrifugation, washed with methanol and deionized water to remove excess Eu^3+^, and dried under vacuum at 80 °C overnight to yield Eu@SUZ−103.

### 2.3. Method Development for LVFX Detection Using Eu@SUZ−103

To evaluate the performance of Eu@SUZ−103 in detecting LVFX, 10 mg of Eu@SUZ−103 was dispersed in 10 mL of ultrapure water, forming a solution with a concentration of 1.0 mg mL^−1^. Subsequently, 0.4 mL of LVFX solution at varying concentrations was added to 0.6 mL of the prepared Eu@SUZ−103 solution. After a 15 min incubation period, the luminescence intensity of Eu@SUZ−103 at 617 nm was measured under an excitation wavelength of 395 nm. To assess the selectivity of Eu@SUZ−103 for LVFX, luminescence responses were also recorded in the presence of various potential interfering species using the same procedure (LVFX solution at a fixed concentration was mixed with the prepared Eu@SUZ−103 solution, with equal volumes of potential interfering species introduced into the mixture under the same conditions). All experiments were conducted at room temperature and performed in triplicate to ensure reproducibility and reliability of the results.

### 2.4. LVFX Monitoring in Serum and Urine

Human serum (from commercial sources) and urine (from author Zhijie Qiu) were used in this study. Briefly, 1 mL of the serum or urine sample was mixed with 1 mL of acetonitrile in a centrifuge tube, followed by vigorous shaking and centrifugation. The resulting supernatant was then diluted 50-fold with ultrapure water and stored at 4 °C for subsequent analyses. LVFX detection was conducted by spiking the appropriately prepared solutions with the target compound.

## 3. Results and Discussion

### 3.1. Structural Characterization of SUZ−103 and Eu@SUZ−103

**Figure 1:**
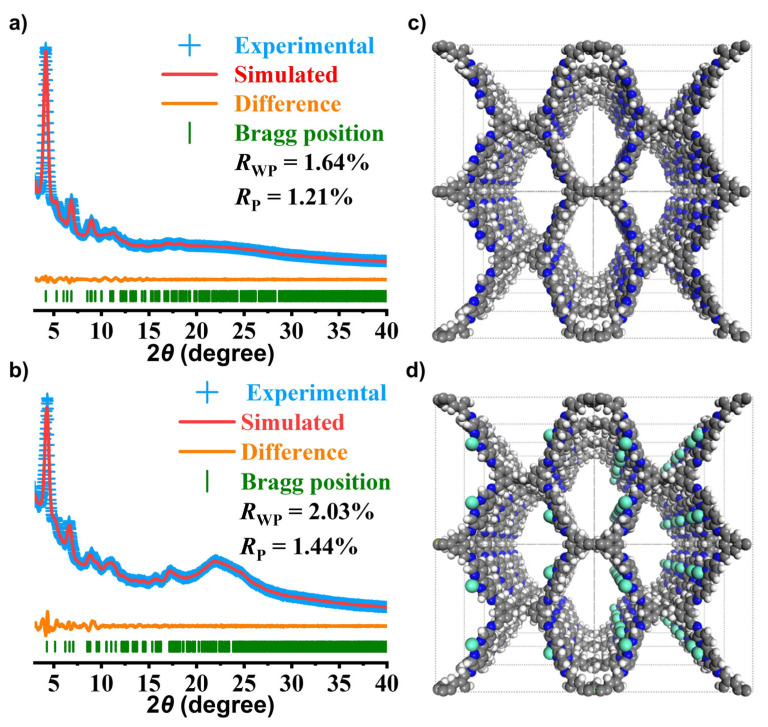
Experimental and refined PXRD patterns of (**a**) SUZ−103 and (**b**) Eu@SUZ−103. Structural representations of (**c**) SUZ−103 and (**d**) Eu@SUZ−103.

The crystal structures of SUZ−103 and Eu@SUZ−103 were confirmed by PXRD measurements in conjunction with structural simulations (Figure 1). After geometrical energy minimization using the Materials Studio software [53] package based on ***bcu*** topology, SUZ−103 unit cell parameters were acquired (a = 13.9557 Å, b = 28.5609 Å, c = 33.0425 Å and α = β = γ = 90). The simulated PXRD patterns were in good agreement with the experimental results. Furthermore, full-profile pattern-matching (Pawley) refinement was applied to the experimental PXRD patterns. Peaks at 4.14°, 5.29°, 6.85°, and 8.95° for SUZ−103 belonged to the (011), (002), (101), and (112) Bragg peaks of space group IMM2 (No. 44). Similarly, the PXRD pattern of Eu@SUZ−103 displayed identical diffraction peaks comparable to those of SUZ−103, demonstrating that the framework retained its main crystal structure and remained highly crystalline following Eu ion modification. The refinement results well matched the observed values, with negligible difference and good agreement factors (R_p_ = 1.21% and R_wp_ = 1.64% for SUZ−103; R_p_ = 1.44% and R_wp_ = 2.03% for Eu@SUZ−103). Based on the above results, the obtained COFs are proposed as the expected architectures featuring the ***bcu*** net (Table S3).

**Figure 2:**
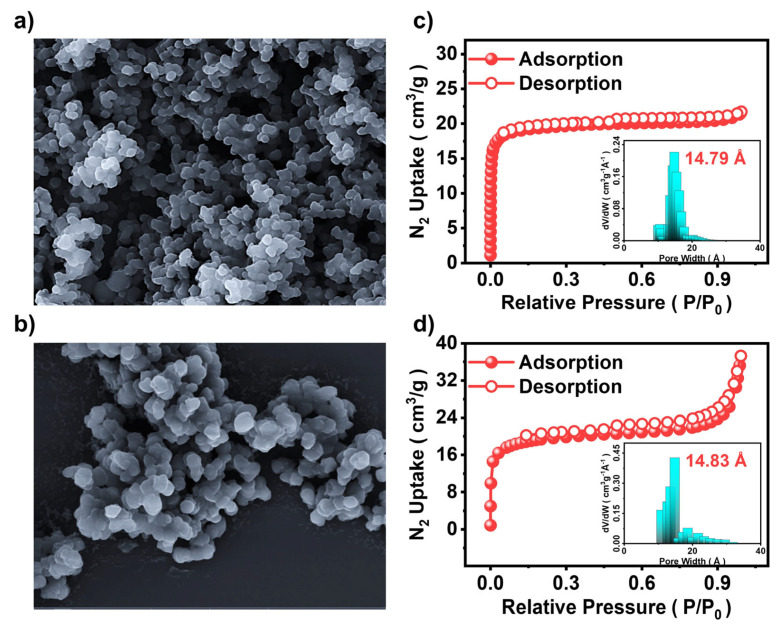
SEM images of (**a**) SUZ−103 and (**b**) Eu@SUZ−103. N_2_ adsorption–desorption isotherms of (**c**) SUZ−103 and (**d**) Eu@SUZ−103 at 77 K (inset: pore width profiles).

A variety of complementary characterization techniques confirmed the successful formation of SUZ−103. In the FT-IR spectra of SUZ−103 (Figure S1), the appearance of new peaks at around 1625 cm^−1^ indicated the formation of imine linkages, while the reduced intensities of the C=O for DPTB-Me and N–H in the Bpy signals at approximately 1700 cm^−1^ and 3357 cm^−1^, respectively, further confirmed the successful transformation [54]. Notably, Eu@SUZ−103 presented similar FT-IR peaks in these regions, indicating that the grafting of Eu did not compromise the overall framework stability. Solid-state ^13^C cross-polarization magic-angle-spinning NMR spectroscopy further verified the presence of C=N bonds represented by the peak at 157 ppm for SUZ−103 (Figure S2) [55]. SEM images (Figure 2a,b) revealed aggregates of spherical nanoparticles for both SUZ−103 and Eu@SUZ−103, indicating no significant morphological changes following Eu ion post-grafting. Thermogravimetric analysis (TGA) under a nitrogen atmosphere (Figure S3) confirmed the high thermal stability of both COFs up to 450 °C. To probe their permanent channels and specific surface areas, nitrogen adsorption–desorption measurements were performed at 77 K. As illustrated in Figure 2c,d, both SUZ−103 and Eu@SUZ−103 exhibited a sharp uptake at low relative pressure (P/P_0_< 0.05), indicative of their microporous nature [55]. Based on the Brunauer–Emmett–Teller (BET) model, the specific surface areas were determined to be 1535.3 m^2^ g^−1^ for SUZ−103 and 1469.4 m^2^ g^−1^ for Eu@SUZ−103. Calculation of pore size distribution via nonlocal density functional theory (NLDFT) revealed micropore widths of 14.79 Å for SUZ−103 and 14.83 Å for Eu@SUZ−103, in good agreement with the proposed structural models.

**Figure 3:**
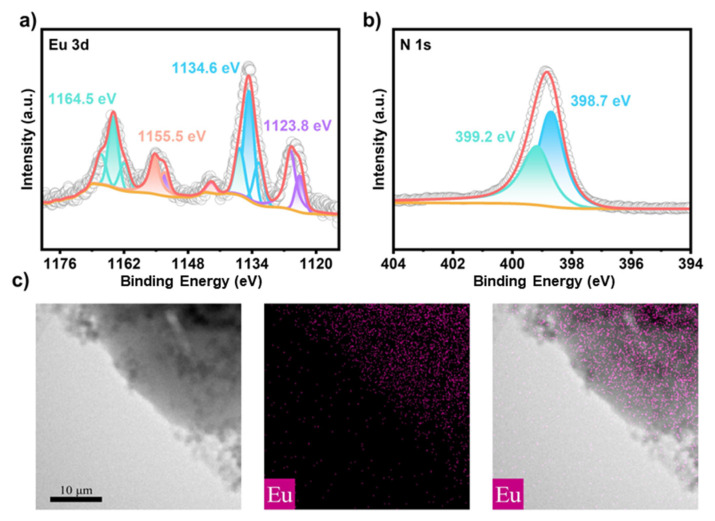
(**a**) High-resolution Eu 3d XPS profiles of Eu@SUZ−103. (**b**) High-resolution N 1s XPS profiles of Eu@SUZ−103. (**c**) EDX patterns of corresponding element maps for Eu@SUZ−103.

X-ray photoelectron spectroscopy (XPS) and energy-dispersive X-ray (EDX) mapping were employed to investigate the chemical composition of Eu@SUZ−103. The XPS survey spectrum (Figure 3a,b) confirmed the successful incorporation of Eu^3+^ ions into the SUZ−103 framework, as evidenced by the characteristic peaks of Eu (III) 3d_3_/_2_ and Eu (III) 3d_5_/_2_ at 1164.5 eV and 1134.6 eV, respectively [56]. Additional peaks at 1155.5 eV and 1123.8 eV corresponded to Eu (II) 3d3_3_/_2_ and Eu (II) 3d_5_/_2_, probably arising from partial reduction during measurement [57]. In the N 1s region (Figure 3b), peaks were observed at 398.7 eV and 399.2 eV, which can be attributed to C=N and C–N bonds, respectively [58,59]. These findings were corroborated by Fourier-transform infrared (FT-IR) spectroscopy (Figure S1), which also indicated the presence of C=N functionalities [60,61]. Furthermore, EDX mapping (Figure 3c) revealed the uniform dispersion of Eu sites throughout the microcrystals, underscoring the effective integration of Eu within the SUZ−103 framework.

### 3.2. Luminescent and Optical Properties of SUZ−103 and Eu@SUZ−103

Photoluminescence (PL) spectroscopy was employed to investigate the optical characteristics of the synthesized materials at room temperature (RT). As depicted in Figure S6, upon excitation at 395 nm, the SUZ−103 host framework revealed a wide emission band that stretches from 475 to 660 nm, culminating in a distinct peak at 513 nm. To enhance the luminescent properties, SUZ−103 was grafted with Eu(acac)_3_ complexes, and the resulting hybrid material was subsequently investigated. Europium complexes were selected for grafting to provide an additional co-ligand, functioning not only as an antenna ligand but also safeguarding the europium ions from quenching by water molecules. Notably, the acetylacetonate (acac) ligand possesses a high level of triplet energy (25,300 cm^−1^), making it a suitable choice for lanthanides, which have high acceptor energy levels. Upon grafting, Eu@SUZ−103 retained the original broad band at 513 nm and also exhibited additional sharp peaks at 617 nm. These peaks are specific to the ^5^D_0_ → ^7^F_k_ (k = 0–4) transitions of Eu^3+^, reflecting the successful embedding of europium into the framework (Figure S8).

### 3.3. Luminescent Detection of LVFX via Eu@ZUC-103

**Figure 4:**
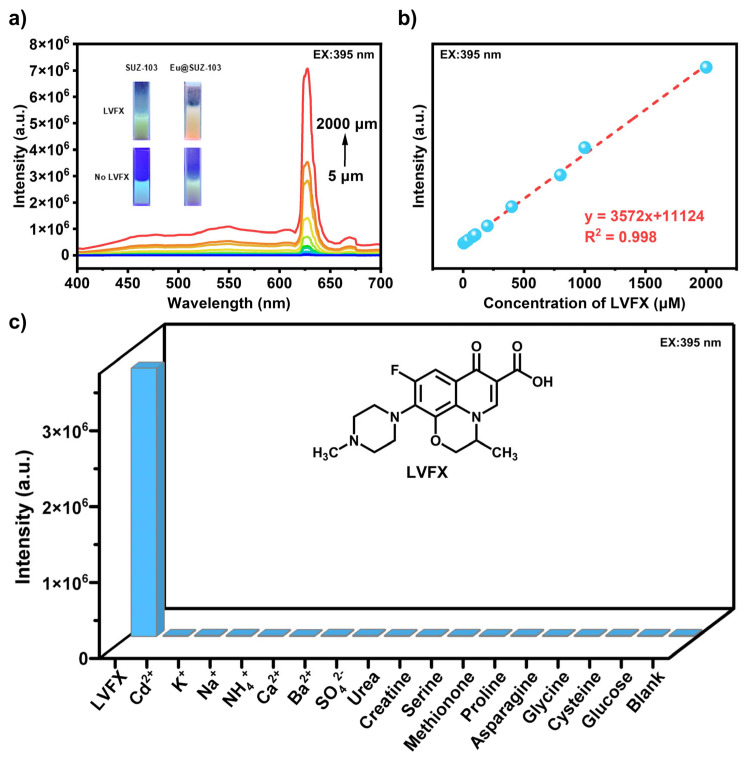
(**a**) Emission spectra of Eu@SUZ−103 were recorded in the presence of varying concentrations of LVFX (5–2000 μM). The inset displays optical images and visual luminescence images of LVFX, Eu@SUZ−103, and Eu@SUZ−103 with LVFX. (**b**) Linear fitting curve of LXFX concentration (5–2000 μM) vs the variation value of the emission peak (617 nm). (**c**) Selectivity of the Eu@SUZ−103 sensor to the substances in body fluids (for more detailed illustrations, please refer to Figure S13). All measurements were performed under an excitation of 395 nm.

We systematically optimized the experimental parameters for detecting LVFX using Eu@SUZ−103, with a focus on understanding the system’s kinetic behavior. Initially, we observed that the luminescence intensity increased with the concentration of Eu@SUZ−103, reaching a maximum at 600 μg/mL (Figure S9), followed by a slight decrease. Kinetic measurements were conducted to assess the response time, and time-course studies showed that the luminescence intensity stabilized after a short period, indicating that equilibrium had been reached (Figure S10). These kinetic results confirmed that the system achieved a stable response within a short period. Based on this, we selected a 15 min incubation time to ensure efficient interaction between LVFX and Eu@SUZ−103 while minimizing any further fluctuations. We also investigated the impact of pH on luminescence. Within a pH range of 6 to 8, the emission spectra of Eu@SUZ−103 exhibited characteristic Eu^3+^ peaks with stable intensity (Figure S11), indicating that the material was able to operate reliably under physiological conditions such as those in human serum and urine. Based on these findings, the optimal conditions for LVFX detection were set at a Eu@SUZ−103 concentration of 600 μg/mL, pH 7, and 15 min incubation. Under these optimal conditions, emission spectra of Eu@SUZ−103 for LVFX detection were recorded. As shown in Figure 4a,b, under 395 nm excitation, the luminescence at 617 nm increased in a dose-dependent manner with rising LVFX concentration, following the calibration curve y = 3572x + 11124 (R^2^ = 0.998). The linear detection range spanned from 5 μM to 2000 μM, with a calculated limit of detection (LOD) of 0.61 μM based on the equation LOD = 3Sd/k (N = 20), where Sd represents the standard deviation of the blank and k is the slope of the regression. The inset in Figure 4a illustrates that under UV irradiation, Eu@SUZ−103 containing LVFX produced red emissions. Recyclability tests (Figure S12) indicated only a slight decrease in fluorescence after five cycles, signifying robust sensor reusability. A comparative performance analysis is provided in Table S2, highlighting the advantages of the Eu@SUZ−103-based sensor.

To explore the sensing mechanism of Eu@SUZ−103 for LVFX detection, the system was analyzed via PXRD, UV-vis spectroscopy, and fluorescence spectroscopy. As shown in Figure S6, the XRD patterns of Eu@SUZ−103 after treatment with LVFX remained almost identical to those of the original Eu@SUZ−103, suggesting that the observed sensing behavior was not due to structural changes. Further analysis of the absorption, excitation, and emission spectra of Eu@SUZ−103 and LVFX (Figures S8, S15 and S16) revealed a significant overlap between the emission spectrum of LVFX and the absorption spectrum of Eu@SUZ−103. This spectral overlap suggests the occurrence of Förster resonance energy transfer (FRET) from LVFX to Eu@SUZ−103 [62]. The specificity of this sensing mechanism is attributed to the β-diketone structure of LVFX, which plays a crucial role in enhancing the luminescence of Eu^3+^. Specifically, the β-diketone moiety in LVFX acts as an efficient “antenna” by absorbing UV light and transferring the excitation energy to Eu^3+^ through the FRET process [63]. Moreover, the triplet state of LVFX in the presence of the COF matrix is likely to be higher than that of Eu^3+^, facilitating efficient transfer of energy from LVFX to Eu^3+^. This energy transfer mechanism explains the significant emission enhancement of Eu@SUZ−103 observed after the addition of LVFX [64].

### 3.4. Evaluation of Selectivity in LVFX Detection

**Figure 5:**
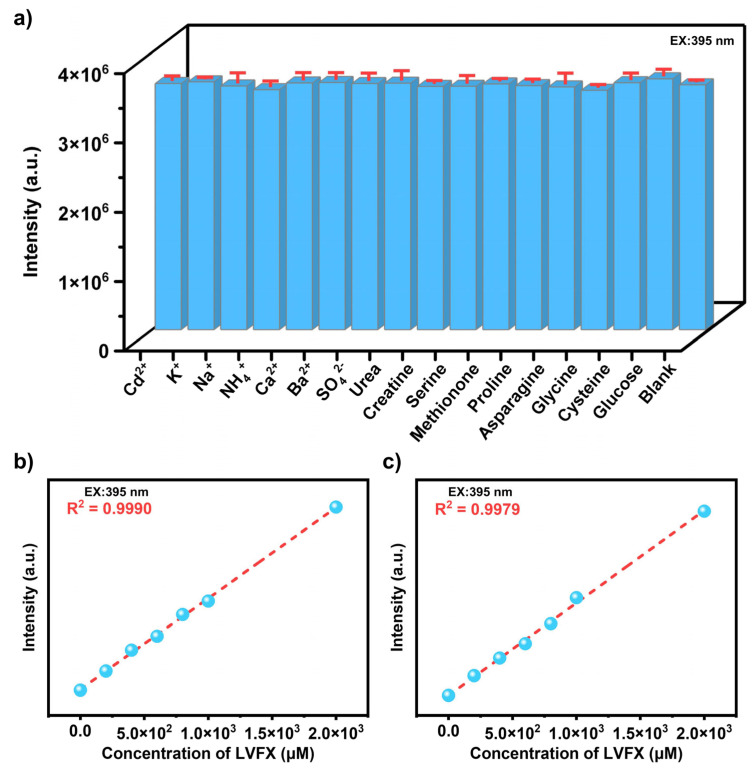
(**a**) Interference study of the Eu@SUZ−103 sensor’s selectivity toward LVFX. Linear fitting curves of LVFX concentration in human (**b**) serum and (**c**) urine samples vs. the emission intensity at 617 nm. All measurements were performed under an excitation of 395 nm.

To assess sensing selectivity, the luminescent response of Eu@SUZ−103 to LVFX was systematically compared against various interferents, including selected amino acids (serine, methionine, proline, asparagine, glycine, and cysteine), metal ions (Cd^2+^, K^+^, Na^+^, NH_4+_, Ca^2+^, Ba^2+^, SO_4_^2−^), and other substances (urea, creatinine, and glucose) commonly found in body fluids (Figure 4c and Figure S13). Among these compounds, only LVFX elicited a marked turn-on luminescence response. To further validate that the specificity could be attributed to the β-diketone structure of quinolone drugs, experiments were conducted by adding Eu@SUZ−103 to mixed solutions of four other quinolone antibiotics that also featured the β-diketone structure. The results revealed that all the antibiotics elicited a turn-on luminescence response, further confirming that the β-diketone played a crucial role in enhancing the luminescent emission of Eu^3+^ (Figure S14). Furthermore, interference experiments were conducted by adding Eu@SUZ−103 to a mixed solution containing LVFX and other substances at a fixed concentration of 600 μM. As shown in Figure 5a, the luminescence intensity remained unchanged, demonstrating that Eu@SUZ−103 is an excellent and highly selective luminescent probe for LVFX detection.

### 3.5. Analysis of Real Biological Samples: Human Serum and Urine

The reliability and practical applicability of the Eu@SUZ−103-based luminescent LVFX sensor were further validated using real serum and urine samples. Linear calibration curves were established by plotting the luminescence intensity at 617 nm against the LVFX concentration in these matrices, as depicted in Figure 5b,c. In both human serum and urine, the sensor exhibited a strong linear response over a concentration range of 5–2000 μM, with correlation coefficients (R^2^) of 0.9990 and 0.9979, respectively. Corresponding limits of detection (LODs) were calculated to be 0.714 μM for serum and 0.55 μM for urine. To assess the method’s accuracy, spike and recovery tests were conducted. As summarized in Table S1, recoveries ranged from 95.93% to 101.89%, with relative standard deviations (RSDs) between 1.27% and 2.49%. These outcomes confirm that the Eu@SUZ−103 sensor offers high sensitivity, reliability, and selectivity, making it a promising tool for LVFX detection in human serum and urine.

## 4. Conclusions

In this study, we successfully synthesized Eu^3+^-grafted covalent organic frameworks (Eu@SUZ−103) featuring an eight-connected bcu topology and demonstrated their efficacy in the selective fluorescence detection of levofloxacin (LVFX). Comprehensive characterization confirmed the robust structural integrity, high crystallinity, and uniform Eu^3+^ distribution within the SUZ−103 framework. Eu@SUZ−103 exhibited exceptional luminescent properties, with a linear detection range of 5–2000 μM and a low limit of detection of 0.61 μM. The sensor displayed remarkable selectivity for LVFX and maintained high performance in complex biological matrices such as human serum and urine, achieving recovery rates between 95.93% and 101.89%. Compared with conventional methods such as colorimetric and electrochemical techniques, the Eu@SUZ−103-based fluorescence sensor is simpler, more sensitive, and offers a wider detection range. making it suitable for clinical and environmental monitoring. Future work will focus on enhancing the framework’s stability and expanding its application to other antibiotics.

## Data Availability

Informed consent was obtained from all subjects involved in the study.

## Supplementary Materials

The following supporting information can be downloaded at: https://www.mdpi.com/article/10.3390/s25072304/s1, Figure S1. FT-IR spectra of SUZ−103 (red), Eu@SUZ−103 (blue), Bpy (orange), DPTB-Me (green). Figure S2. Solid state 13C NMR of SUZ−103. Figure S3. TGA curve of SUZ−103 (red), Eu@SUZ−103 (blue). Figure S4. BET plot of SUZ−103 calculated from N2 adsorption isotherm at 77 K. Figure S5. BET plot of Eu@SUZ−103 calculated from N2 adsorption isotherm at 77 K. Figure S6. Emission spectra of SUZ−103. Figure S7. Emission spectra of Eu@SUZ−103. Figure S8. Optimization of Eu@SUZ−103 concentration. Figure S9. Optimization of Eu@SUZ−103 incubation time. Figure S10. Emission spectra of Eu@SUZ−103 at different pH of 6, 7 and 8. Figure S11. Recyclability of Eu@SUZ−103 for LVFX detection in water. Figure S12. The selectivity of the Eu@SUZ−103 sensor to the substances in body fluids (The detailed version of Figure 4C)). Table S1. Determination of LVFX in human serum and urine samples (n = 3). Table S2. The comparison for determination of LEVX based on different sensing platforms. Table S3. Unit cell parameters and fractional atomic coordinates for SUZ−103 calculated based on the bcu net. References [65,66,67,68,69,70,71,72,73,74,75,76] are cited in the Supplementary Materials.
