# Supplementary material for: Novel Europium-Grafted 3D Covalent Organic Framework for Selective and Sensitive Fluorescence-Enhanced Detection of Levofloxacin
Source: Sensors (Basel). 2025 Apr 4;25(7):2304. doi: 10.3390/s25072304 (PMC11991030; doi:10.3390/s25072304)
Supplement: Supplementary file 1 [file sensors-25-02304-s001.zip › sensors-3472238-supplementary.pdf]

Supplementary information

Novel Europium-Grafted 3D Covalent Organic Framework for Selective and Sensitive Fluorescence-Enhanced Detection of Levofloxacin

Junyi Zhao [1,4,‡], Chao Zhang [5,‡], Zhijie Qiu [3], Zerong Zhang [3], Xiaorou Lin [3], Shibin Huang [3], Jianfeng Zhang [3], Jingpeng Wu [3], Li Liao [1,2]* and, Rui Wang [3]*

Junyi Zhao 1,2 , †, Chao Zhang 3, †, Zhijie Qiu 4, Zerong Zhang 5, Xiaorou Lin 5, Shibin Huang 5, Jianfeng Zhang 5, Jingpeng Wu 5, Li Liao 1,6, and Rui Wang 5,**

1 *Northeast Guangdong Key Laboratory of New Functional Materials, School of Chemistry and Environment, Jiaying University, Meizhou 514015, China; junyi98@mail.ustc.edu.cn*

2 *Hefei National Research Center for Physical Sciences at the Microscale, School of Physical Sciences, University of Science and Technology of China, Hefei 230026, China*

3 *The Third Affiliated Hospital of Anhui Medical University (The First People's Hospital of Hefei), Hefei 230001, China; chao.zhang@ahu.edu.cn.*

4 *Guangdong Weipu Testing Technology Co., Ltd., Guangzhou 510275, China ; qzjieaa@gmail.com*

5 *State Key Laboratory of Inorganic Synthesis and Preparative Chemistry, Jilin University, Changchun 130012, China; zerongzhang0314@gmail.com (Z.Z.); 202430700765@mail.scut.edu.cn (X.L.); shibinhuang666779@outlook.com (S.H.); zjf1930468484@outlook.com (J.Z.); wujingpeng17613@outlook.com(J.W.)*

6 *School of Chemical Engineering and Technology, College of Chemistry, GBRCE for Functional Molecular Engineering, IGCME, Sun Yat-sen University, Zhuhai 519082, China*

* *Correspondence: liaoli3@mail.sysu.edu.cn (L.L.); rwang19@mails.jlu.edu.cn (R.W.)*

† *These authors contributed equally to this work.*

Table of contents

Section S1	Materials and characterization	S3-S9
Section S2	FT-IR spectra	S10
Section S3	Solid-state ^{13}C NMR spectra	S10
Section S4	TGA curves	S11
Section S5	Nitrogen adsorption	S11-12
Section S6	Stability	S12
Section S7	Fluorescence Test	S13-S17
Section S8	Determination of LVFX	S18
Section S9	Unit cell parameters and fractional atomic coordinates	S19-20

Section S1. Materials and characterization

S1.1 Materials

All starting materials and solvents, unless otherwise noted, were obtained from J&K Scientific Ltd （Beijing，China） and used without further purification. All products were isolated and handled under nitrogen using either glovebox or Schlenk line techniques.

S1.2 Instruments

Solid-state ^{13}C NMR spectra were recorded on an AVIII 500 MHz solid-state NMR spectrometer. The FTIR spectra (KBr) were obtained using a SHIMADZU IRAffinity-1 Fourier transform infrared spectrophotometer. A SHIMADZU UV-2450 spectrophotometer was used for all absorbance measurements. Thermogravimetric analysis (TGA) was recorded on a SHIMADZU DTG-60 thermal analyzer under N_2. The operational range of the instrument was from 30 °C to 800 °C at a heating rate of 10 °C min^{-1} and N_2 flow rate of 30 mL min^{-1}. PXRD data were collected on a PANalytical B.V. Empyrean powder diffractometer using a Cu Kα source (λ = 1.5418 Å) over the range of 2ϑ = 2.0–40.0° with a step size of 0.02° and 2 s per step. The sorption isotherm for N_2 was measured using a Quantachrome Autosorb-IQ analyzer with ultra-high-purity gas (99.999% purity). To estimate pore size distributions for SUZ-103 and Eu@SUZ-103, nonlocal density functional theory (NLDFT) was applied to analyze the N_2 isotherm on the basis of the model of N_2@77K on carbon with slit pores and the non-negative regularization method. To obtain scanning electron microscopy (SEM) images, JEOL JSM-6700 scanning electron microscope was applied. Transmission electron microscopy (TEM) images were obtained on a JEM-2100 transmission electron microscope. All fluorescence measurements were performed using a FluoroMax Plus spectrofluorometer.

S1.3 Synthesis of 5-(4,4,5,5-tetramethyl-1,3,2-dioxaborolan-2-yl)isophthalaldehyde (TDI)

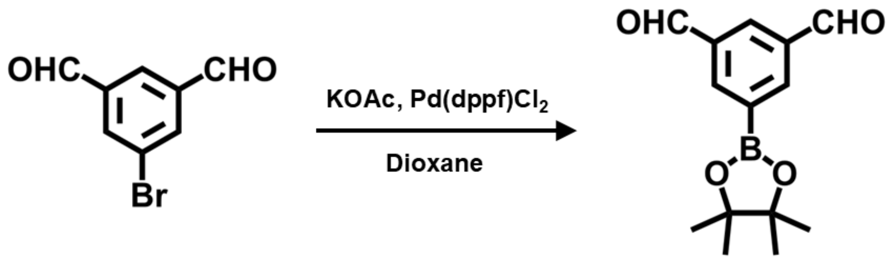

Synthesis of TDI: TDI was synthesized following a similar method to that described in the literature [65]. Under Ar atmosphere, a reaction mixture containing 5-bromoisophthalaldehyde (3.0 g, 14.1 mmol), bis(pinacolato)diboron (4.5 g, 17.7 mmol), KOAc (4.14 g, 42.2 mmol), Pd(dppf)Cl_2 (307 mg, 0.43 mmol), and dioxane (60 mL) was heated to 95 °C and stirred for 24 h. After cooling to room temperature, the mixture was filtered through diatomite and the filtrate was evaporated to dryness after being extracted with EA, washed with brine, and evaporated under reduced pressure, giving the crude product. The crude compound was dissolved in DCM and purified by silica gel column chromatography with PE/DCM (1/3) as eluent to afford the TDI as an off-white solid. ^{1}H NMR (400 MHz, $CDCl_3$) δ 10.13 (s, 2H), 8.55 (d, 2H), 8.46 (t, 1H), 1.39 (s, 12H).^{13}C NMR (101 MHz, $CDCl_3$) δ 191.2, 141.3, 136.3, 132.1, 84.7, 77.4, 77.1, 76.8, 24.8.

S1.4 Synthesis of 4',5'-bis(3,5-diformylphenyl)-3',6'-dimethyl-[1,1':2',1"-terphenyl]-3,3'',5,5"-tetracarbaldehyde (DPTB-Me)

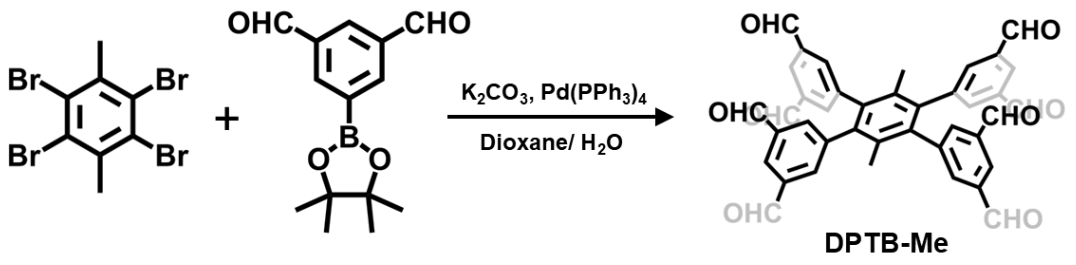

Synthesis of DPTB-Me: DPTB-Me was synthesized following a similar method to that described in the literature [65]. A mixture containing 1,2,4,5-tetrabromo-3,6-dimethylbenzene (843 mg, 2 mmol), TDI (3.55 g, 10 mmol), K_2CO_3 (2.2 g, 16 mmol), $Pd(PPh_3)_4$ (230 mmg, 0.2 mmol) was added to dioxane (30 mL) and H_2O (5 mL) under Ar atmosphere. This reaction was heated to 95 °C and stirred for 36 h. After cooling to room temperature, the mixture was filtered without further purification to afford the final product DPTB-Me as an off-white solid. ^1H NMR (400 MHz, DMSO) δ 10.00 (s, 8H), 8.18 (s, 4H), 7.99 (s, 8H), 1.84 (s, 6H). ^{13}C NMR (400 MHz, DMSO) δ 188.4, 139.5, 138.0, 133.1, 130.9, 124.6, 122.4, 24.8.

S1.5 Synthesis of 2,2'-bipyridine-5,5'-diamine (Bpy)

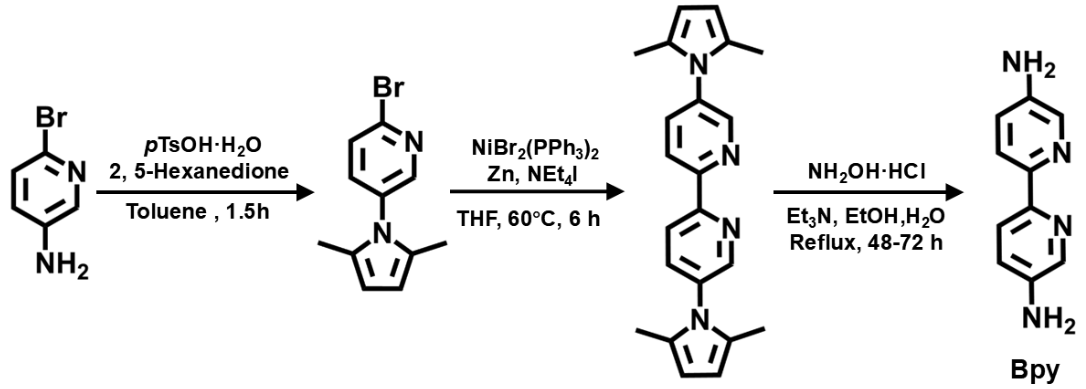

Step 1: Synthesis of 1-1-(2-Bromopyridine-5-yl)-2,5-dimethyl-1H-pyrrole: 1-1-(2-Bromopyridine-5-yl)-2,5-dimethyl-1H-pyrrole was synthesized following a similar method to that described in the literature [66]. To a 100 mL flask was added 5-amino-2-bromopyridine (5.917 g, 29 mmol), 50 mL toluene, 4.1 mL hexane-2,5-dione (3.97 g, 34.8 mmol), and p-toluenesulfonic acid monohydrate (276 mg, 1.5 mmol). This mixture was heated in a Dean − Stark apparatus for 1.5 h until completion of the reaction (LC-MS). After cooling to room temperature, the mixture was quenched with a saturated aqueous solution of $NaHCO_3$ (50 mL) and separated. The organic layer was washed with water (50 mL) and dried over $MgSO_4$. After removing the solvent by rotary evaporation, the resultant crude product was purified by column chromatography (SiO_2, PE/EtOAc: 20/1) to yield the product 1-1-(2-Bromopyridine-5-yl)-2,5-dimethyl-1H-pyrrole as a pale pink solid. ^{1}H-NMR (400 MHz, $CDCl_3$): δ 8.29 (d, 1H), 7.61 (d, 1H), 7.43 (d, 1H), 5.94 (s, 2H), 2.04 (s, 6H). ^{13}C-NMR (100 MHz, $CDCl_3$): δ 149.6, 140.9, 138.1, 135.2, 129.0, 128.5, 107.3, 13.1.

Step 2: Synthesis of 5,5'-Bis(2,5-dimethyl-1H-pyrrol-1-yl)-2,2'-bipyridine: 5,5'-Bis(2,5-dimethyl-1H-pyrrol-1-yl)-2,2'-bipyridine was synthesized following a similar method to that described in the literature [67-68]. In a 250 mL two-necked flask, activated zinc powder (2.64 g, 40.4 mmol), tetraethylammonium iodide (6.1 g, 23.7 mmol), and $NiBr_2(PPh_3)_2$ (3.53 g, 4.8 mmol) were suspended in 25 mL dry THF and stirred at 60 °C under argon for one hour. 1-1-(2-Bromopyridine-5-yl)-2,5-dimethyl-1H-pyrrole (5.96 g, 23.7 mmol) was dissolved in dry THF (60 mL) and slowly added to this mixture with an addition funnel and the reaction mixture was stirred 6 h, after which it was allowed to cool down to room temperature. Then, concentrated ammonia (25%, 100 mL), water (50 mL), and CH_2Cl_2 (100 mL) were added and this mixture was stirred for 15 min, after which it was filtered over celite. The phases were separated and the aqueous phase was extracted twice with 100 mL of CH_2Cl_2. The combined

organic phases were evaporated under reduced pressure and the crude product was purified by column chromatography (SiO_2, hexane/EtOAc/Et3N: 10/1/0.05) as eluent to obtain the product 5,5'-Bis(2,5-dimethyl-1H-pyrrol-1-yl)-2,2'-bipyridine as a light yellow solid. ^{1}H-NMR (400 MHz, $CDCl_3$): δ 8.57-8.60 (m, 4H), 7.72 (d, 2H), 5.98 (s, 4H), 2.10 (s, 12H). ^{13}C-NMR (100 MHz, $CDCl_3$): δ 154.5, 148.6, 136.5, 135.9, 129.1, 121.5, 107.0, 13.2.

Step 3: Synthesis of Bpy: Bpy was synthesized following a similar method to that described in the literature [69]. To a 100 mL flask were added 5,5'-bis(2,5-dimethyl-1H-pyrrole)-2,2'-bipyridine (2.23 g, 6.53 mmol), hydroxylamine hydrochloride (13.61 g, 196 mol), 20 mL H_2O, 50 mL absolute EtOH, and 8 mL triethylamine. This mixture was refluxed for 24 h, after which another 30 eq. of hydroxylamine hydrochloride and 4 mL triethylamine were added and the reaction was continued for another 24 h. If LC-MS analysis then indicated complete conversion of the starting material, the reaction was stopped, if not, another 30 eq. of NH_2OH.HCl and 8 mL of triethylamine were added and the mixture was stirred for another 24 h. Upon completion, the reacted mixture was allowed to cool down to room temperature and then, 30 mL of 3 N HCl was added followed by 100 mL of EtOH. The mixture was put in the freezer overnight and filtered to obtain the hydrochloride salt of the product as an orange powder. This powder was dissolved in water and sodium hydroxide solution (3N) was added until the mixture was highly alkaline (pH 11). This was then extracted multiple times with dichloromethane, dried over $MgSO_4$, and filtered. The solvent was removed under reduced pressure to obtain the product Bpy as a light yellow solid. It can also (more conveniently) be obtained in a similar yield by allowing the product to precipitate from the basic solution. The precipitate can then be filtered off and washed with water, followed by extensive drying under vacuum to remove residual water. ^{1}H-NMR (400 MHz, DMSO-d_6): δ 7.91 (d, 2H), 7.86 (d, 2H), 6.95 (d, 2H), 5.32 (s, 4H). ^{13}C-NMR (100 MHz, DMSO-d_6): δ 144.8, 143.8, 135.0, 120.6, 119.1.

S1.6 Synthesis of SUZ-103

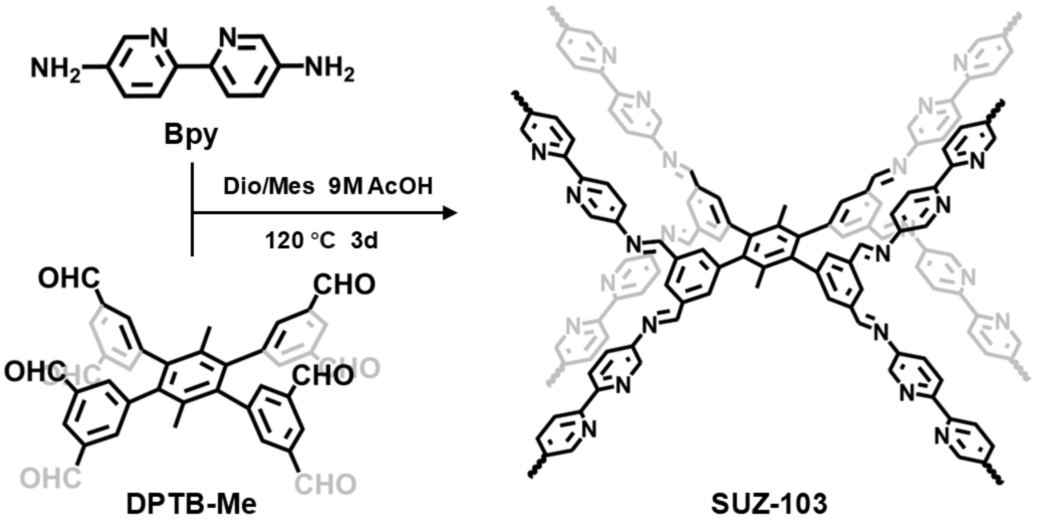

Synthesis of SUZ-103: In a Pyrex tube (volume: approximately 20.0 mL, body length: 18.0 cm, neck length: 9.0 cm), DPTB-Me (0.018 mmol, 11.42 mg) and 2,2'-bipyridine-5,5'-diamine (Bpy) (0.072 mmol, 13.41 mg) were weighed and added with 0.2 mL of Dio, 0.8 mL of mesitylene, and 0.2 mL of acetic acid (9 M). The Pyrex tube was flash frozen in a liquid nitrogen bath, evacuated to an internal pressure of approximately 19.0 mbar, and flame-sealed, reducing the total length by about 10.0 cm. Once warmed to room temperature, the tube was placed in an oven at 120 °C for 72 h. The resulting precipitate was filtered and exhaustively washed by Soxhlet extraction with acetone for 48 h. The resulting precipitate was separated by centrifugation and then washed with DMF (10 mL x 3 times) followed by dioxane (10 mL x 3 times) and finally cleaned in THF via Soxhlet extraction for 24 h. The final solid was dried at 80 °C under vacuum for 12 hours, yielding approximately 18 mg of SUZ-103 as a light yellow solid.

S1.7 Synthesis of Eu@SUZ-103

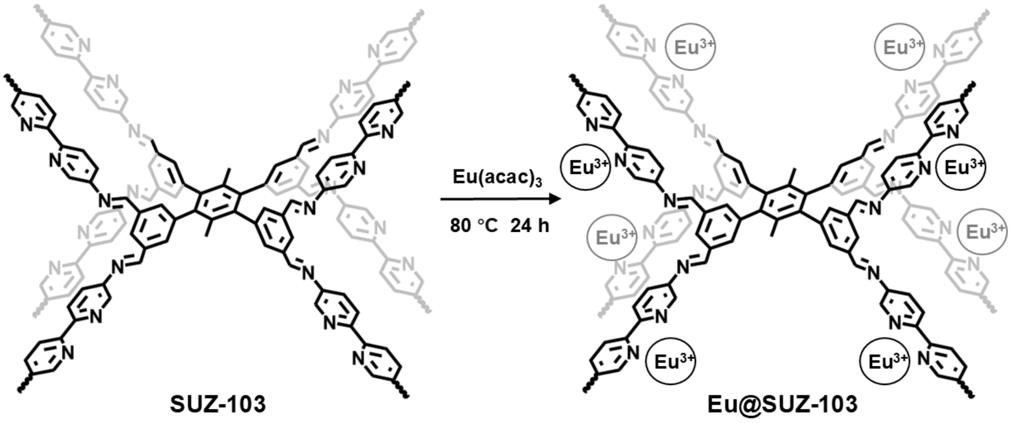

Synthesis of Eu@SUZ-103: Freshly prepared SUZ-103 (70 mg) was added to a vial of 10 mL of a lanthanide salt $Eu(acac)_3$ solution (deionized water as the solvent, 0.01 M) and soaked for 24 h at 80 °C. The product was collected via centrifugation and washed with deionized water three times to remove the excess of Eu^{3+} physically absorbed on the surface. The product was then dried at 80 °C under vacuum overnight to afford Eu@SUZ-103 as a dark yellow powder. To quantify the metal ion content, X-ray fluorescence analysis (XRF) was performed, revealing that the mass fraction of Eu^{3+} in the COF material was approximately 27%. Comparing this value with the bulk composition of the COF, we estimate that approximately 80% of the bipyridine fragments were coordinated to Eu^{3+} ions.

Section S2. FT-IR spectra

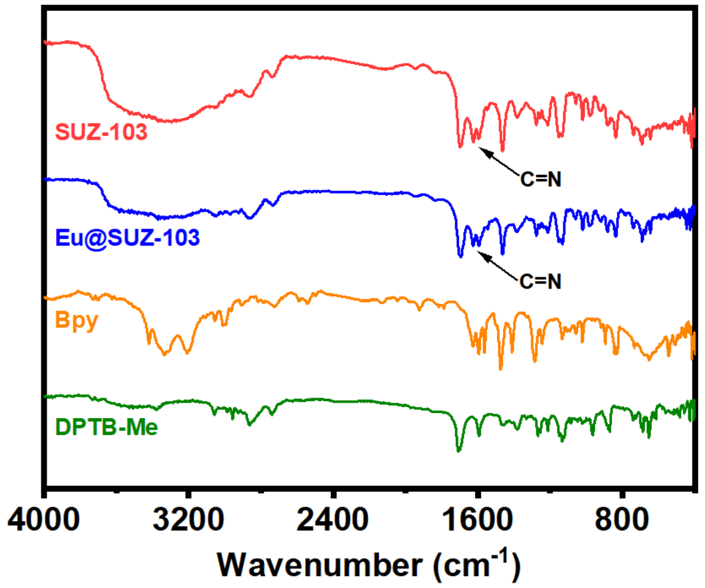

Figure S1. FT-IR spectra of SUZ-103 (red), Eu@SUZ-103 (blue), Bpy (orange), DPTB-Me (green).

Section S3. Solid-state ^{13}C NMR spectra

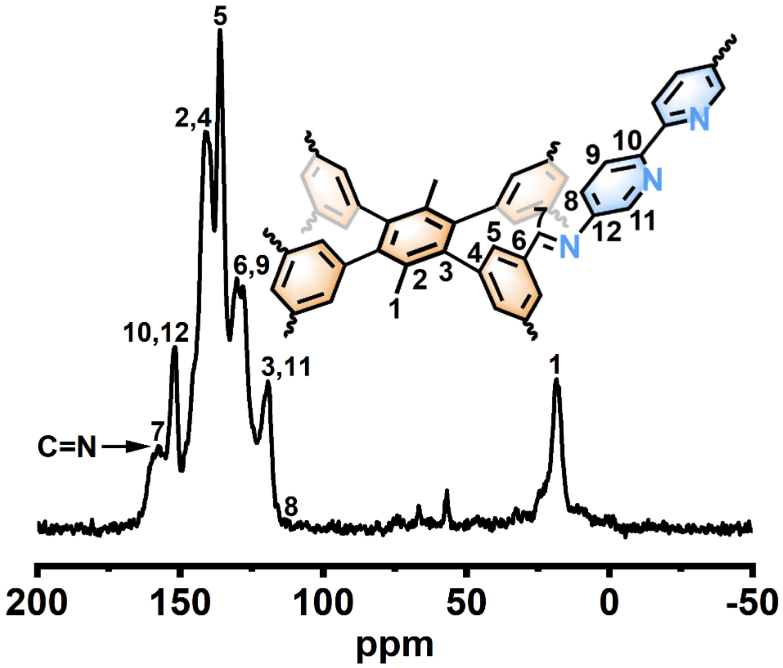

Figure S2. Solid state ^{13}C NMR of SUZ-103.

Section S4. TGA curves

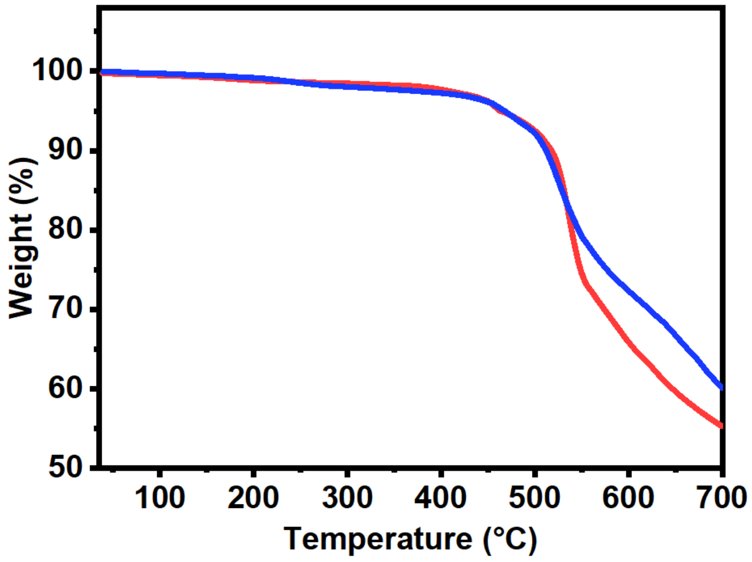

Figure S3. TGA curve of SUZ-103 (red), Eu@SUZ-103 (blue).

Section S5. Nitrogen adsorption

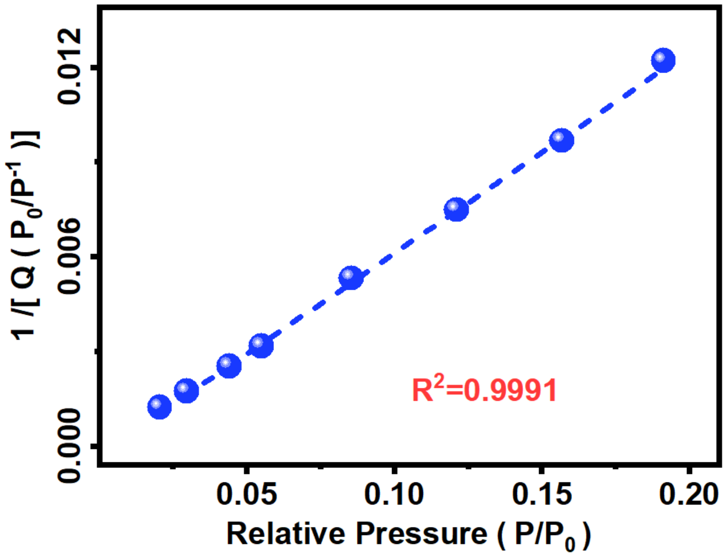

Figure S4. BET plot of SUZ-103 calculated from N_2 adsorption isotherm at 77 K.

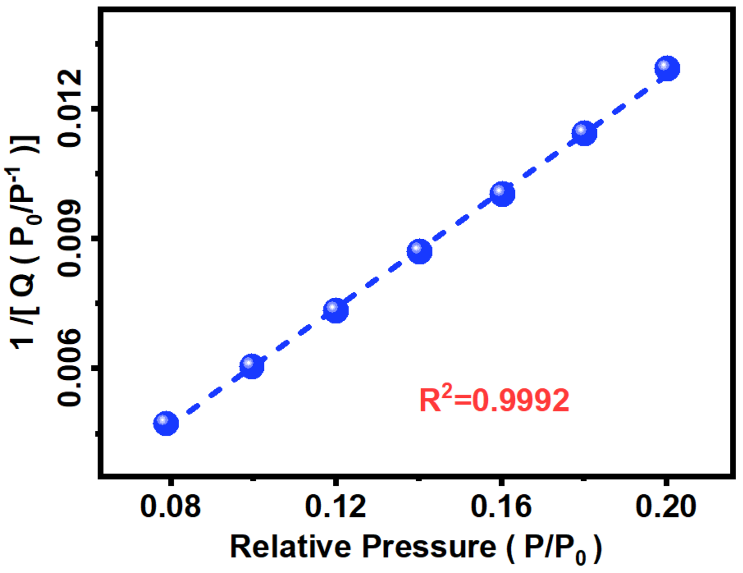

Figure S5. BET plot of Eu@SUZ-103 calculated from N_2 adsorption isotherm at 77 K.

Section S6. Stability

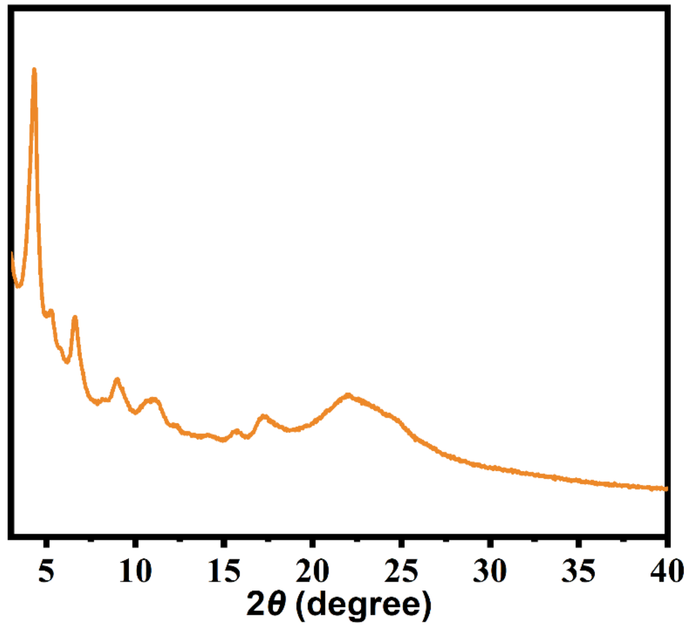

Figure S6. PXRD patterns of Eu@SUZ-103 after treatment with LVFX.

Section S7. Fluorescence Test

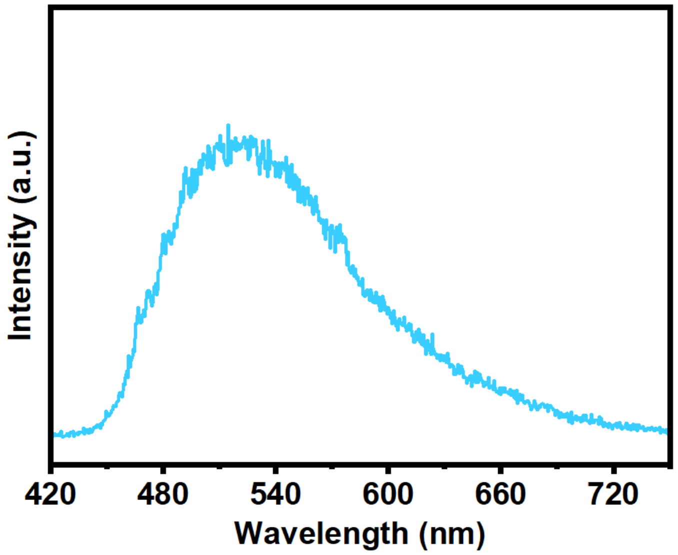

Figure S7. Emission spectra of SUZ-103.

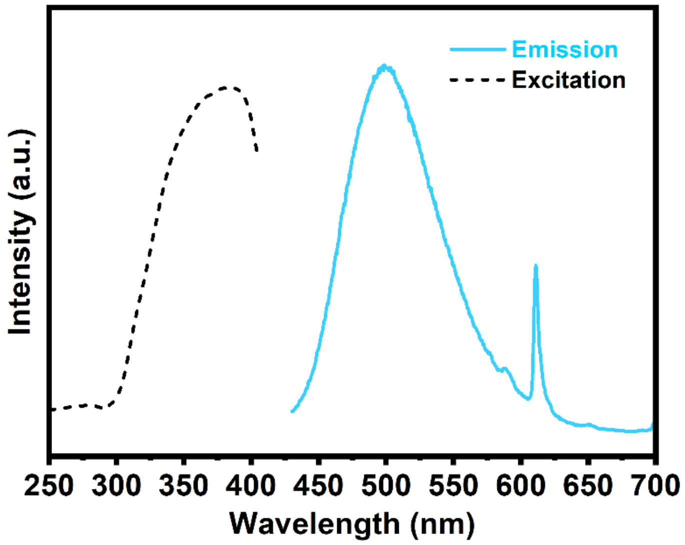

Figure S8. Emission spectra and excitation spectra of Eu@SUZ-103.

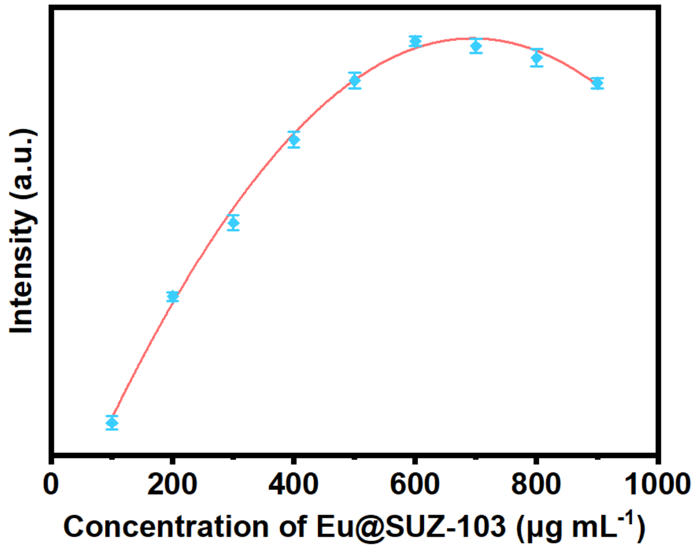

Figure S9. Optimization of Eu@SUZ-103 concentration.

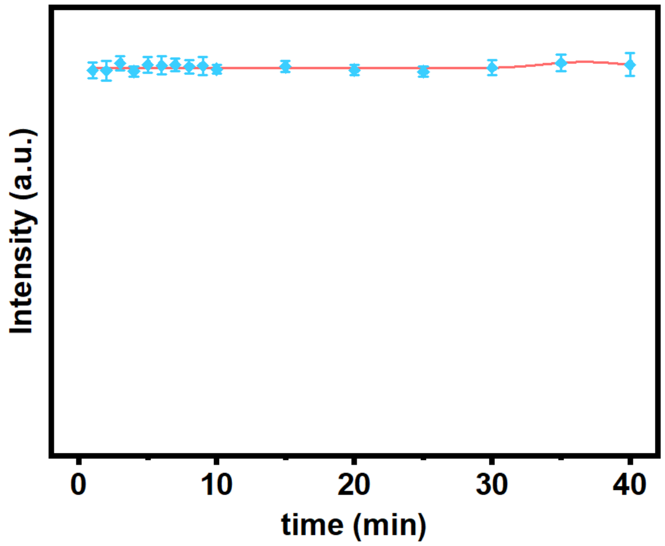

Figure S10. Optimization of Eu@SUZ-103 incubation time.

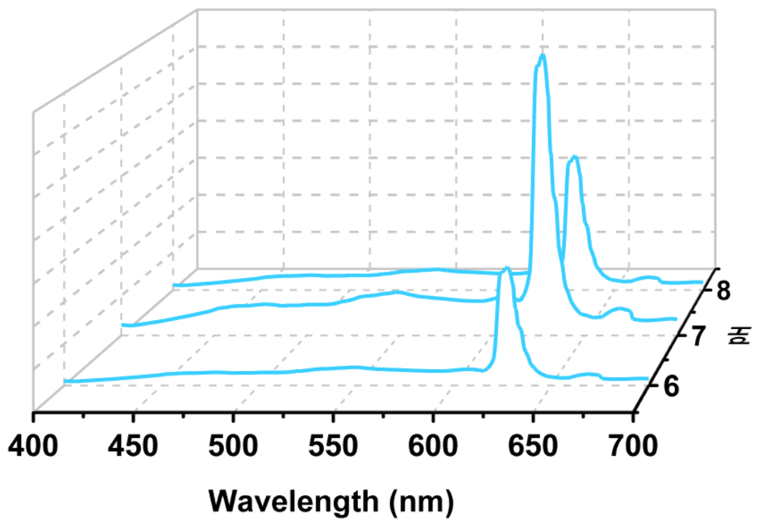

Figure S11. Emission spectra of Eu@SUZ-103 at pH 6, 7, and 8.

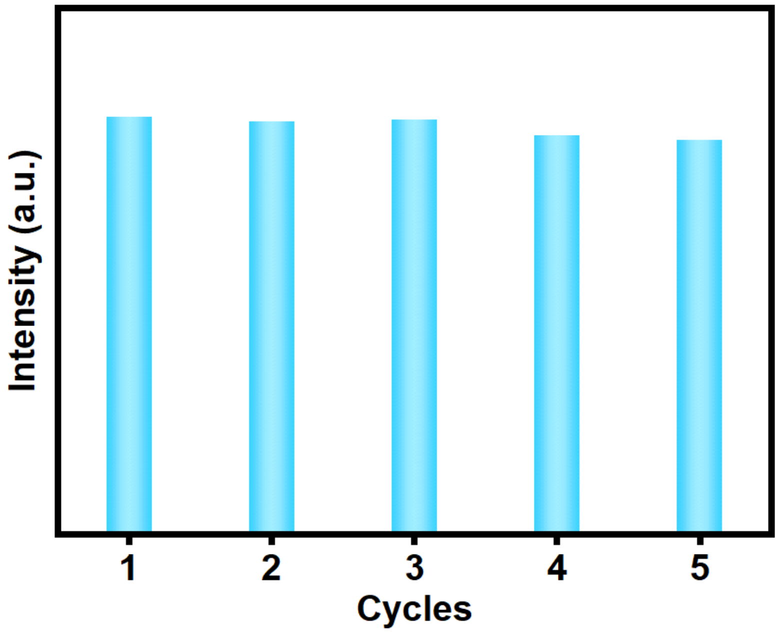

Figure S12. Rrecyclability of Eu@SUZ-103 for LVFX detection in water.

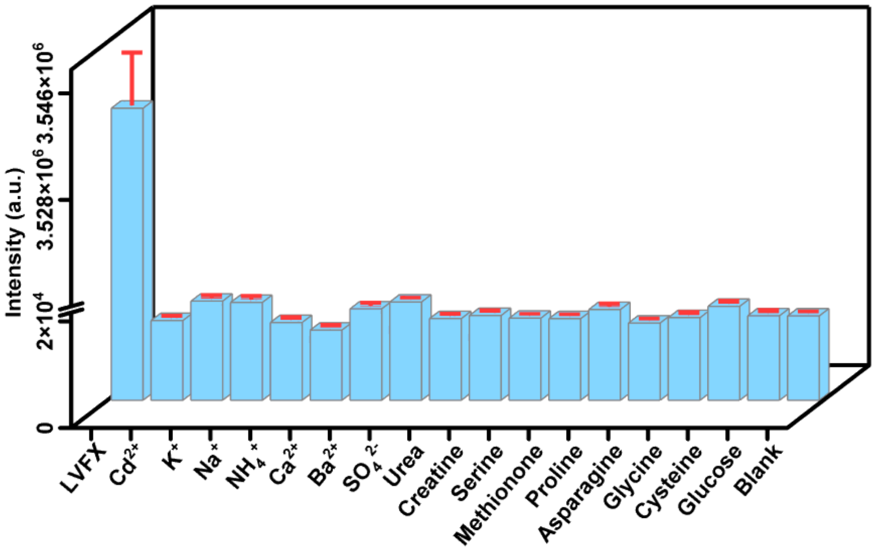

Figure S13. The selectivity of the Eu@SUZ-103 sensor to the substances in body fluids (a detailed version of Figure 4c).

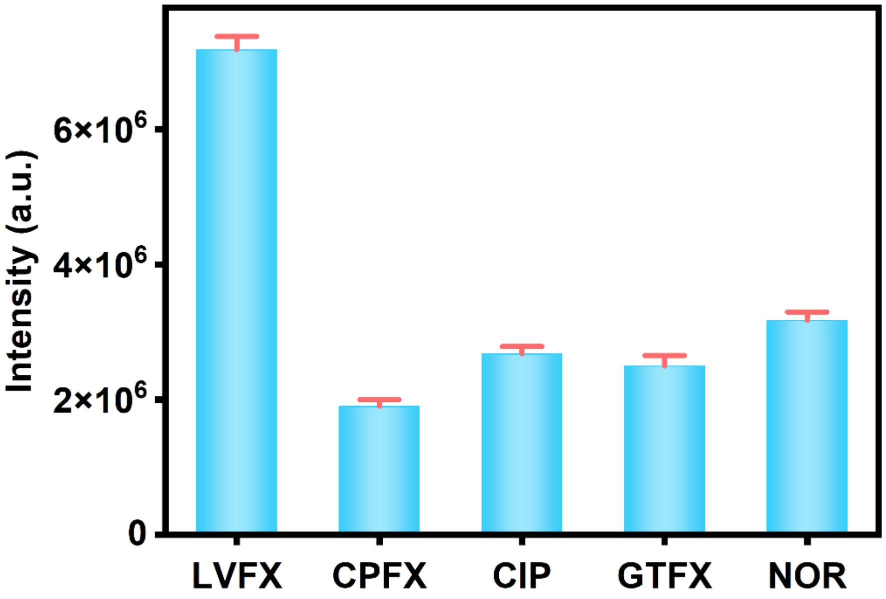

Figure S14. Optical responses of Eu@SUZ-103 to a range of quinolone drugs (the conditions for quinolone drug detection were set at an Eu@SUZ-103 concentration of 600 μg/mL, pH 7, and 15 minutes incubation).

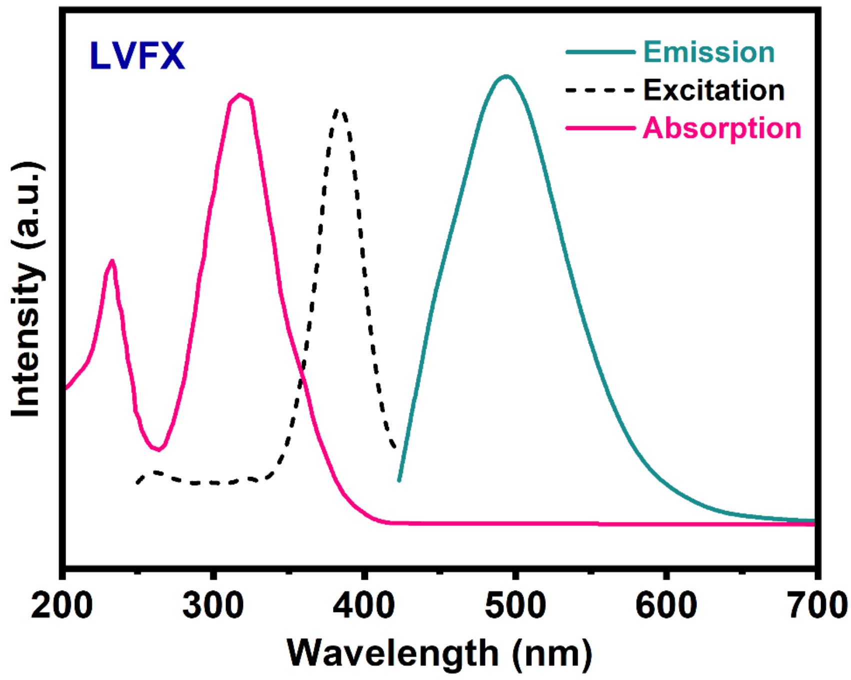

Figure S15. UV-Vis absorption spectrum, excitation and emission spectra of LVFX.

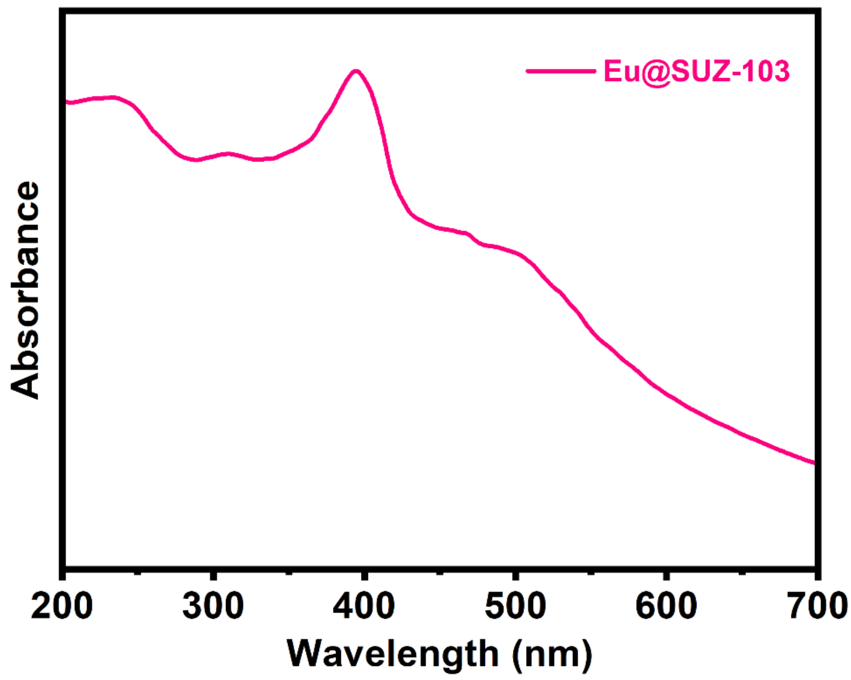

Figure S16. UV-Vis absorption spectrum of Eu@SUZ-103.

Section S8. Determination of LVFX

Table S1. Determination of LVFX in human serum and urine samples (n = 3).

Sample	Spiked (μM)	Average±SD	RSD (%)	Recovery (%)
Serum	0	Not detected		
	10	9.59 ± 0.14	1.41	95.93
	100	101.89 ± 2.31	2.27	101.89
	500	502.49 ± 8.62	1.72	100.5
Urine	0	Not detected		
	10	9.63 ± 0.24	2.49	96.33
	100	99.66 ± 1.54	1.55	99.66
	500	496.68 ± 6.33	1.27	99.34

*Average was determined from three replicates.

Table S2. The comparison for determination of LEVX based on different sensing platforms.

Methods	Materials	Detection range (μM)	LOD (μM)	Ref.
**Fluorescence**	**Eu@SUZ-103**	**5-2000**	**0.73**	**This work**
Fluorescence	Eu@CON	2-800	0.51	[38]
Fluorescence	Tb@TFP-EB	5-240	1.26	[71]
Fluorescence	Eu@TpPa-1	1-1000	0.2	[72]
Colorimetric	Methanol	8.30-22.14	0.69	[73]
Electrochemical	MWCNT@Ni-BTC	2-100	0.2	[74]
Electrochemical	3D-Gr@Al_2O_3	2-100	0.128	[75]
Electrochemical	Au/PDDA/rGO/GCE	10-800	3.90	[76]
Spectrofluorimetric	Ammonium vanadate	41.5-110.7	0.72	[77]
Fluorescence	UiO-66-F_4	30-68	0.1	[11]

Section S9. Unit cell parameters and fractional atomic coordinates

Table S3. Unit cell parameters and fractional atomic coordinates for SUZ-103 calculated based on the **bcu** net.

Space group	*IMM2*		
Calculated unit cell	a = 13.9557 Å, b = 28.5609 Å, c = 33.0425 Å, $\alpha = \beta = \gamma$ = 90°		
Measured unit cell	a = 13.9626 Å, b = 28.6621 Å, c = 33.1324 Å, $\alpha = \beta = \gamma$ = 90°		
Pawley refinement	R_P = 1.21 %, R_{WP} = 1.64 %		
atoms	x	y	z
C1	-0.82796	0.04932	-0.89314
C2	-0.82134	0.0986	-0.86932
N3	-0.6691	0.10041	-0.15003
C4	-0.14436	0.30103	-0.65366
C5	-0.13294	0.35347	-0.66937
C6	-0.1313	0.04941	-0.10363
C7	-0.37182	0.62443	-0.26614
C8	-0.82098	0.76934	-0.78463
C9	-0.88779	0.83406	-0.73888
C10	-0.8639	0.78045	-0.74828
C11	-0.29017	0.4506	-0.62512
C12	-0.24837	0.39927	-0.63567
N13	-0.32345	0.40687	-0.33086
C14	-0.80554	0.18926	-0.81187
C15	-0.83251	0.13615	-0.80291
C16	-0.62073	0.45057	-0.42761
C17	-0.6056	0.86053	-0.20873
C18	-0.13828	0.74385	-0.67885
C19	-0.10094	0.68396	-0.73405
C20	-0.12137	0.73613	-0.71974
H21	-0.83071	0.13791	-0.88337
H22	-0.16551	0.29543	-0.623
H23	-0.10411	0.08746	-0.0927
H24	-0.39216	0.58324	-0.25913
H25	-0.79808	0.72874	-0.79183
H27	-0.28794	0.36244	-0.63589
H28	-0.772	0.19876	-0.83956
H29	-0.59746	0.41254	-0.44027
H30	-0.59673	0.90108	-0.22084
H32	-0.08533	0.67723	-0.76526
H33	-0.78213	-0.03681	-0.00026
C34	-0.95371	0	-0.98197
C35	-0.75114	0	-0.13601
C36	-0.90478	0	-0.0911

H37	-0.69034	0	-0.15303
C38	-0.04619	0	-0.05391
C39	-0.20106	0	-0.87704
C40	-0.09527	0	-0.9446
H41	-0.23938	0	-0.84965
C42	-0.90636	0	-0.01778
C43	-0.80565	0	-0.01629
H44	-0.77255	0	-0.04567
